# 

**Published:** 1891-02

**Authors:** 


### CONTENTS.

ORIGINAL COMMUNICATIONS—

Rupture of the Membrana Tympani by Injection of Warm Water into the Auditory Canal for the Removal of Impacted Cerumen. By S. Latimer Phillips, M. D., of Savannah, Ga ..... 707

Concerning Dr. Howell's Case. By E. H. Martin, M. D., Hillhouse, Miss ..... 712

Dr. Martin's Report of Eleven Cases of "So-called" Malarial Hematuria ..... 715

One Hundred Consecutive Cases of Skin Disease, II. By M. B. Hutchins, M. D ..... 725

SOCIETY REPORTS—

Gynecological and Obstetrical Society of Baltimore, Md.... 729

Allegheny County Medical Society ..... 740

Atlanta Society of Medicine ..... 751

The American Electro-Therapeutic Association ..... 754

CORRESPONDENCE—

Our New York Letter ..... 755

Letter from R. C. Gercke ..... 760

BOOK REVIEWS ..... 761

Books Received ..... 762

EDITORIAL—

Koch's "Lymph" in Lupus ..... 763

The Grady Hospital ..... 764

The Latest Fad ..... 764

Resolutions of Respect ..... 765

Items ..... 766–770

Index to Advertisements ..... xi

Reading Notices ..... x

Premium List ..... xxxvi-xxxvii

